# Will Human-Animal Chimeras Cause Moral Confusion? Exploring Public Attitudes

**DOI:** 10.1007/s11673-024-10413-4

**Published:** 2025-08-12

**Authors:** Katrien Devolder, Joshua Rottman, Qinyu Xiao, Guy Kahane, Lucius Caviola, Lauren Yip, Nadira S. Faber

**Affiliations:** 1 Uehiro Oxford Institute, University of Oxford, Littlegate House, St Ebbes, Oxford, OX1 1PT UK; 2 Department of Psychology, Franklin & Marshall College, Lancaster, PA USA; 3 Department of Occupational, Economic, and Social Psychology, Faculty of Psychology, University of Vienna, Vienna, Austria; 4 Global Priorities Institute, University of Oxford, Oxford, UK; 5 Vincent Hospital, Melbourne, Australia; 6 Department of Psychology, University of Bremen, Bremen, Germany

**Keywords:** Human-animal chimeras, Moral status, Xenotransplantation, Moral confusion

## Abstract

Recent medical research involving human-monkey chimeras, human brain organoids in rats, and the transplantation of a gene-edited pig heart and gene-edited pig kidneys in living human beings have intensified the debate about whether we should create human-animal chimeras for biomedical purposes and, if so, how we should treat them. Influential views in the debate frequently appeal to assumptions regarding how people will react to such chimeras. It has, for example, been argued that the most important objection against creating such chimeras is that this will result in inexorable moral confusion about species boundaries and will, as a result, threaten the social order. But is this indeed the case? We conducted three empirical studies to examine laypeople’s views on the creation and treatment of various types of human-animal chimeras. Our studies indicate that laypeople find typical cases of xenotransplantation (i.e., the transplantation of an animal organ into a human patient) morally unproblematic. They assign the same moral status to humans with animal organs as to non-chimeric humans. By contrast, they sometimes (but not always) assign slightly higher moral status to animals with human organs than to non-chimeric animals. Overall, however, there is little indication of chimera technology blurring the line between humans and animals, and thus of the technology causing moral confusion.

**Supplementary Information:**

The online version contains supplementary material available at 10.1007/s11673-024-10413-4.

## Introduction

Recent biomedical research involving human-monkey chimeras, human brain organoids in rats, and the transplantation of a gene-edited pig heart and gene-edited pig kidneys in living human beings have re-ignited the debate about whether we should create human–non-human animal chimeras for biomedical purposes, and if so, how we should treat such chimeras (Streiffer 2005; Eberl and Ballard 2009; Devolder et al. 2019; Kataoka et al. 2023).

Chimeras are organisms that consist of cells or tissues from different genetic origins. The cells or tissues in a chimera can belong to the same or different species. Here, we are interested in chimeras consisting of cells or tissues from both non-human animals (for reasons of brevity, just “animals” henceforth) and humans.

Scientists have been creating human-animal chimeras for biomedical research for decades (Behringer 2007). The primary aim of these endeavours is to gain insights into human biology and diseases by utilizing animal models (usually mice and rats) that contain human cells or tissues. By introducing human cells into animal embryos or inserting them into specific tissues during different stages of development (including postnatal development), researchers aim to study various aspects of human development, disease progression, and potential therapeutic interventions.

More recently, with advancements in genome editing and stem cell research, scientists are exploring the potential of human-animal chimeras to create improved models for studying human diseases, including brain diseases, and to address the worldwide shortage of donor organs. This kind of research has re-ignited the debate about the ethics of creating human-animal chimeras.

Three scientific projects in particular have attracted the attention of bioethicists and the media.

First, human brain organoids have been successfully transplanted into rat brains (Revah et al. 2022). Human brain organoids are three-dimensional models developed from stem cells that mimic certain aspects of the human brain’s structure and function. After a human brain organoid was successfully transplanted into a damaged rat brain, it integrated with the rat’s visual system and responded to flashing light stimuli. The hope is that this sort of research will offer scientists a better understanding of human neural circuits and allow them to test therapeutic interventions for neurological disorders in humans.

Second, between 2022 and 2024, the heart and kidneys of genetically modified pigs were successfully transplanted into human patients who had no other medical options (Kotz and Seiler 2022). In January 2022, a genetically modified pig heart was transplanted into a fifty-seven-year-old man in the end stage of heart disease. He survived for two months with the organ, which was considered a good short-term result. In 2024, two patients received a genetically modified pig kidney transplant. A sixty-two-year-old man with end-stage kidney disease lived for two months with the organ (Mallapaty and Kozlov 2024). The second transplant was performed in a fifty-four-year-old woman suffering from heart and end-stage kidney disease. The kidney was removed after forty-seven days due to loss of function, and the patient died two months later (Lewis 2024). All these xenotransplantation procedures (i.e., the transplantation of a primarily animal organ to a human) used pigs that were genetically modified to incorporate human genes to reduce the risk of organ rejection and virus transmission. They mark a major step towards using pigs as organ donors for human patients as a solution to the worldwide problem of donor organ shortage.

The third project that attracted attention and ignited debate was carried out to learn more about the possibility of using pigs as organ donors for humans. In 2021, a team of scientists used a technique called *interspecies blastocyst complementation* to inject human pluripotent stem cells into macaque embryos (Tan et al. 2021). The experiment was considered a success because it was the first time a significant proportion of human cells were present in developing human-monkey chimeric embryos. One of the aims of these experiments is to develop strategies to improve the integration of human cells in evolutionarily more distant animal species, like pigs, in the hope that, ultimately, it will be possible to use them as hosts to grow cells, tissues, and organs (henceforth simply “organs”) for transplantation into humans. The ultimate goal is to genetically modify a pig embryo to prevent it from growing a specific organ. This would create a developmental niche within which injected human pluripotent stem cells could be expected to generate the missing organ (Matsunari et al. 2013). Most of the resulting human-pig chimera’s tissues would be comprised of a mix of (mainly) pig and human cells, but the targeted organ would mostly, or even entirely, consist of human cells (Rashid et al. 2014). Furthermore, if the patient’s induced pluripotent stem (IPS) cells were used to create the chimeric animal, the organ would consist of cells originating from the patient, which obviates the need for lifelong immunosuppression after organ transplantation. This sort of transplantation is referred to as exotransplantation (where a primarily human organ grown in an animal is transplanted into a human being). Pigs are preferred over primates as a source of organs, as their organs are the right size, they are less likely to pass on infection, and they breed rapidly. Their use is generally also considered legally and morally less problematic than the use of primates, as laypeople tend to assign pigs a particularly low moral status compared to other animals (Caviola et al. 2021).

The ethics of the creation and use of human-animal chimeras has been fiercely debated, both in public outlets and the bioethics literature. Influential views in the debate frequently appeal to assumptions regarding how people will react to such chimeras. It has, for example, been argued that the most important objection against creating such chimeras is that this will result in inexorable moral confusion about species boundaries and will, as a result, threaten established social practices (Stout 1988; Robert and Baylis 2003). But is this indeed the case? Several empirical studies have previously examined public attitudes toward xenotransplantation. A meta-analysis (Mitchell et al. 2020) and multiple focused studies have investigated attitudes among medical providers, patients, parents, and different demographic groups (Padilla, Hurst, et al. 2020a, Padilla, Sorabella, et al. 2020b, Padilla, Rhodes, et al. 2021a, Padilla, Hurst, et al. 2021; Hurst et al. 2021). While this research provides valuable insights into public acceptance of xenotransplantation procedures, it has not specifically addressed whether such procedures create moral confusion about species boundaries or affect perceptions of moral status—the focus of our current investigation.

How some media, policymakers, and scientists have been reacting in alarm to the idea of human-animal chimeras at least gives initial support to the hypothesis that the general public indeed finds or will find chimeras and their creation deeply disturbing (Brock 2017; Davis 2019; Lovelace 2021). Some have therefore stressed the need for further discussion regarding the ethics of crossing species boundaries (Garry, Caplan, and Garry 2022). To contribute to this debate, we conducted three empirical studies to inquire about laypeople’s attitudes regarding xenotransplantation, and the moral status of various types of human-animal chimeras. We will refer to these studies as Study 1 (attitudes on xenotransplantation), Study 2 (attitudes on the moral status of human-pig chimeras specifically), and Study 3 (attitudes on the moral status of human-animal chimeras more generally).

We begin by summarizing what we refer to as the “Moral Confusion Argument” and by developing what we think is the most plausible interpretation of it, motivating our three studies along the way. We then discuss our study results and show how they cast doubt on the Moral Confusion Argument. We end by discussing other interesting implications of our studies and by proposing some future directions of research.

## Inexorable Moral Confusion?

It has been claimed that the main concern associated with the creation of human-animal chimeras is that it would result in “inexorable moral confusion in our existing relationships with nonhuman animals and in our future relationships with part-human hybrids and chimeras” (Robert and Baylis 2003, 9). According to Robert and Baylis, whose discussion of this has been influential in the debate, the problem is that people generally accord moral status to humans based on their species membership (i.e., on their “humanness”), whereas to animals based on how useful they are to humans (i.e., their instrumental value). The creation of human-animal chimeras (who are arguably neither fully human nor fully animal) will, so the argument goes, confuse people “as there is no clear way of understanding (or even imagining) our moral obligations to these beings” if they can no longer draw a sharp line between humans and animals (Robert and Baylis 2003, 9). Worst of all, by amounting to puzzling intermediate cases that are neither just human nor just animal, it is claimed human-animal chimeras would make us doubt the assumption that humanness is necessary or sufficient for full moral status. Human-animal chimeras will thus threaten our privileged position on top of the “moral status ladder,” as well as our social institutions since these depend on a clear moral distinction between humans and animals.

Note that Robert and Baylis do not themselves endorse a speciesist view on moral status—that is, the view that species membership is what determines moral status. Their claim relies on the idea that most people are, however, speciesist. Although the speciesist view has been widely criticized in the normative literature (Steinbock 1978; Singer 1990, 2016; Tooley 1998; McMahan 2008), it is indeed prevalent in the wider (Western and adult) population (Caviola et al. 2019; McGuire et al. 2025). Several recent studies support Robert and Baylis’s assumption that people indeed attribute a higher moral status to humans simply in virtue of their being human, even after targets’ differences in cognitive capacities and sentience are controlled for (Caviola et al. 2022). But what is unclear is whether people, even if they hold such “speciesist” views, would indeed be inexorably morally confused by the creation of human-animal chimeras and whether this would be perceived as a threat to our privileged position on the moral standing ladder and even to our social institutions. On the one hand, chimeras might indeed be thought to occupy an uncanny moral location between humans and animals, thus confoundingly splintering the bright line that typically allows non-humans to be treated quite differently from humans, in line with Robert and Baylis’s prediction. But another possibility is that the perceived moral status of chimeras would remain equivalent to the moral status of non-chimeric animals, thus sidestepping any moral confusion amongst the general public.

To determine the extent to which human-animal chimeras might cause moral confusion, we asked our study participants (Study 2) about their views on the moral status of pigs and rats with one or several human organs and that of humans with one or several pig organs. We also asked them about their views on the moral status of human-animal chimeras in general (Study 3) and on the blurring of species boundaries in the context of xenotransplantation (Study 1).

According to Robert and Baylis, human-animal chimeras with potentially enhanced cognitive capacities will be considered particularly problematic because most people think that human beings are superior to animals “by virtue of the human capacity for reason and language,” and the problem with human-animal chimeras with enhanced cognitive capacities is that they, in particular, will blur the boundary between humans and “unreasoning” animals (Robert and Baylis 2003, 9).

To find out laypeople’s views about human-animal chimeras with potentially enhanced cognitive capacities, we asked participants (Study 2) whether they thought a pig with human neurons (which could potentially affect its cognitive abilities) has a higher, equal, or lower moral status compared to a normal pig. We also asked other participants (Study 3) whether they thought that rats with human brain tissue had a different moral status to normal rats, where we made clear that the human brain tissue was not expected to alter the cognitive abilities of the rat. This allowed for a strong test of whether biological material per se would impact moral status beyond potential changes in psychological qualities (e.g., intelligence and emotional experience), which have been previously shown to have some relevance for lay perceptions of moral status (Gray et al. 2012; Caviola et al. 2022).

Our results thus shed important light on public perception of human-animal chimeras and their moral significance and, more specifically, allow us to directly test the Moral Confusion Argument against the creation of human-animal chimeras.

Some philosophers have argued that moral confusion arising from human-animal chimeras could be beneficial, as it might force us to critically examine our potentially problematic moral categories regarding species boundaries (Pietrzykowski 2018; Koplin and Savulescu 2019; Koplin and Wilkinson 2019). While we acknowledge the value of challenging established moral frameworks, this paper has a more focused aim: to empirically test the assumption that human-animal chimeras would indeed cause moral confusion. This is important groundwork for both practical policy decisions and philosophical discussions, including about the desirability of such moral category disruption.

Finally, our focus is on testing the moral confusion argument in the context of currently feasible forms of human-animal chimeras, particularly those being developed for xenotransplantation. While more extreme forms of chimeras are conceivable, we investigate public attitudes toward the types that are most relevant to current scientific developments and ethical debates.

## Empirical Studies

We conducted three studies to shed light on the moral confusion argument. In Study 1, we probed laypeople’s attitudes towards the xenotransplantation technology. In Study 2, we investigated both the moral status assigned to humans when they carry pig organs *and* the moral status assigned to pigs when they carry human organs. Study 3 then examined whether implanting human brain tissue into non-human animals leads people to confer these animals with elevated moral status. For all three studies, relevant ethical guidelines were followed, and the research was approved by the University of Oxford Research Ethics Committee (reference numbers R57715/RE001 and R56657/RE006). Participants provided informed consent and were financially compensated for their time, with a flat fee that was anticipated to be at or above the minimum wage. We preregistered Study 1 (https://osf.io/wcf8x) and Study 3 (https://osf.io/pxjkf) on the Open Science Framework. In all studies, participants completed questionnaires through Qualtrics survey software.

## Study 1: Attitudes on Xenotransplantation

### Method

Participants (*N* = 500 adults from the United States) were recruited on Amazon Mechanical Turk, an online crowdsourcing site, via CloudResearch. Based on a preregistered criterion, one participant was excluded from analysis for reporting that they did not take the study seriously. Additionally, there was a comprehension check at the beginning of the study, and participants who failed after two attempts (*n* = 21) were not allowed to proceed and were replaced. There were thus 499 participants in the final sample (mean age = 40.8; 275 men, 219 women, 3 non-binary/transgender/third-gender, 2 preferred not to disclose their gender).

Participants were probed about their moral judgements of transplanting animal hearts and kidneys to human recipients, as well as about factors that could have contributed to these moral judgements. Ratings were made on seven-point scales. For moral judgements, these scales ranged from −3 (very wrong) to 3 (very right), with ratings near 0 indicating moral ambivalence. In assessing participants’ reasons for their moral judgements, scales ranged from 1 (strongly disagree) to 7 (strongly agree). We asked a wide range of questions, including a series of validated scale measures; for brevity, we present only the most relevant details here. For a full description of the methods, please see the Supplementary Materials.

## Results

Our findings consistently indicated that lay people generally think xenotransplantation is morally unproblematic. Participants did not morally condemn transplanting animal organs into humans in general (*n* = 499, *M* = 0.85, *SD* = 1.56), and hearts (*n* = 242, *M* = 0.79, *SD* = 1.71) or kidneys (*n* = 257, *M* = 0.90, *SD* = 1.53) in particular. One-sample *t*-tests indicated that participants’ moral judgements were significantly above the midpoint for general moral judgements, *t*(498) = 12.13, *p* < 0.001, *d* = 0.54; for judgements about heart transplants, *t*(241) = 7.19, *p* < 0.001, *d* = 0.46; and for judgements about kidney transplants, *t*(256) = 9.48, *p* < 0.001, *d* = 0.59. One-sample *t*-test against the neutrality midpoint showed that participants disagreed that the procedure made them feel nauseated (*M* = 2.88, *SD* = 1.80, *p* < 0.001, *d* =  −0.62), that it is against God’s will (*M* = 2.71, *SD* = 1.72, *p* < 0.001, *d* =  −0.75), that it makes the recipient human impure (*M* = 2.34, *SD* = 1.59, *p* < 0.001, *d* =  −1.04), that it is unsafe (*M* = 3.37, *SD* = 1.49, *p* < 0.001, *d* =  −0.42), or––most relevant to the focus of this paper––that it blurs the distinction between animals and humans (*M* = 2.94, *SD* = 1.83, *p* < 0.001, *d* =  −0.58).^1^ Additionally, participants disagreed that xenotransplantation would make a recipient less human (*M* = 2.22, *SD* = 1.60, *p* < 0.001, *d* =  −1.11), more similar to the animal the organ came from (*M* = 2.21, *SD* = 1.54, *p* < 0.001, *d* =  −1.17), or less morally valuable (*M* = 1.85, *SD* = 1.34, *p* < 0.001, *d* =  −1.61); they also disagreed that xenotransplantation would lead to changes in recipients’ thinking and reasoning (*M* = 2.10, *SD* = 1.49, *p* < 0.001, *d* =  −1.27), make the recipient less responsible for their behaviours (*M* = 1.82, *SD* = 1.38, *p* < 0.001, *d* =  −1.58), or cause the recipient to have more animal-like sensory and emotional experiences (*M* = 2.08, *SD* = 1.52, *p* < 0.001, *d* =  −1.26).

Overall, the results of this study indicate that people do not find typical cases of xenotransplantation to be immoral, to impact the moral status of organ recipients, or to introduce any inexorable confusion. However, perhaps focusing on the xenotransplantation of animal organs into human recipients sidesteps the potentially greater moral consequences implicated in creating chimeric animals with human organs. We, therefore, focus on this latter issue in our next two studies.

## Study 2: Attitudes on the Moral Status of Human-Pig Chimeras

### Method

Participants (*N* = 249 adults from the United Kingdom) were recruited via Prolific, an online crowdsourcing site. We excluded sixty-seven participants who failed at least one attention check, leaving 182 participants for the final analyses (mean age = 37.3, 48 men, 132 women).

Participants rated how much they morally valued various human-pig chimeras when compared to a standard pig and a standard human. To ensure that intrinsic rather than instrumental value was being assessed, participants were asked whether pigs with human organs mattered morally more or less than a standard pig “in and of itself, irrespective of its value to humans,” and whether humans with pig organs mattered morally more or less than a standard human “in and of itself, irrespective of any harm to pigs.” Participants were asked to answer on a comparative scale where 1 was ‘Much less’, 4 was ‘Exactly the same’ (as a standard pig or a standard human), and 7 was ‘Much more’. For a fuller description of the methods, including other questions that were asked, please see the Supplementary Materials.

## Results

In line with the results of Study 1, we found no significant differences in moral values accorded to humans with pig organs when compared to a standard human. A one-sample *t*-test against the midpoint 4 showed that—compared to a standard human—participants did not assign less moral value to a human who was described as carrying a pig heart (*M* = 4.06, *SD* = 0.73; *p* = 0.46, *d* = 0.08), carrying a pig liver (*M* = 4.04, *SD* = 0.72; *p* = 0.65, *d* = 0.05), or carrying brain cells from a pig (*M* = 4.05, *SD* = 1.09; *p* = 0.69, *d* = 0.04). Hence, xenotransplantation did not reduce a human’s moral status in the participants’ view. We found, however, a tendency for people to assign a slightly higher moral value to a pig—compared to a standard pig—when the pig was described to carry a human heart (*M* = 4.25, *SD* = 0.08; *p* = 0.01, *d* = 0.29), a human liver (*M* = 4.21, *SD* = 0.79; *p* = 0.01, *d* = 0.26), or human brain cells (*M* = 4.66, *SD* = 1.08; *p* < 0.001, *d* = 0.61). Note, however, that while reaching statistical significance, 72.7% of participants chose the mid-point of 4, which indicates “exactly the same” moral values.

Our results of Study 2 indicate that while animal organs do not make a human being less human in their moral status, there seems to be a possibility for animals to be somewhat enhanced in their moral status for carrying human organs. However, in this study, we asked participants very explicitly to directly compare a chimeric pig to a standard pig in its moral status, and still, we found only a small (yet significant) rise above the mid-point. We therefore conducted a third study to replicate and further investigate this effect, specifically beyond the case of pigs. In this third study, we focused on brain cells since these yielded the strongest effect in Study 2.

## Study 3: Attitudes on the Moral Status of Human-Animal Chimeras

### Method

Participants (*N* = 339 adults from the United States) were recruited through Prolific. Based on a preregistered decision, two participants were excluded for admitting to not paying attention, six participants were excluded for failing an open-ended attention check, and one participant was excluded due to an indication that they had already completed the study. Additionally, there was a comprehension check at the beginning of the study, and respondents who failed (*n* = 45) were not allowed to participate and were replaced. This left a final sample of 330 participants for analysis (mean age = 41.7; 166 men, 155 women, 6 non-binary/transgender/third-gender, 3 preferred not to disclose their gender).

Participants first read a description of human brain tissue implants in rats and answered some basic comprehension checks. They then answered questions assessing their perceptions of the moral status of chimeric animals, on scales with options ranging from 1–7, with 4 (the midpoint) indicating similarity between chimeric and non-chimeric animals. Also using 7-point scales, participants rated their beliefs about whether implanting human brain tissue blurs the line between humans and animals, and their resistance to these scientific technologies. They also indicated how “human” various chimeras would be. Once again, we report only the most essential methods and findings here; for a description of additional measures and results, please see the Supplementary Materials.

## Results

The results were partly in line with those of Study 2. Like in Study 2, there was a slight tendency for people to think that having a human brain tissue implant makes an animal morally worth more than a “normal” animal (*M* = 4.20, *SD* = 0.77), which is statistically higher than the scale midpoint, *t*(329) = 4.79, *p* < 0.001, *d* = 0.26. The effect size was again rather small, however, and the modal response was clearly the scale midpoint (81.5% of participants). In addition, participants expressed more resistance to eating bacon from a pig with human brain tissue (*M* = 4.27, *SD* = 2.01) than bacon from a “normal” pig (*M* = 5.46, *SD* = 1.78); this difference was statistically significant, *t*(329) = 12.07, *p* < 0.001, *d* = 0.66. Although this is only speculation, this latter tendency could be partly driven by people’s general resistance to unnatural foods (e.g., GMOs) rather than any concern relating to altered moral status (Scott et al. 2018).

However, on our remaining items, we found evidence that human brain tissue implants do not meaningfully impact people’s perceptions of chimeric animals’ moral status. Beyond merely uncovering non-significant effects (in which there is not sufficient reason to reject the null hypothesis), we conducted equivalence tests to test for statistically significant evidence of the *lack* of effects.^2^ We found that participants thought that animals with human brain tissue should have the same amount of moral rights as “normal” (non-chimeric) animals (*M* = 4.01, *SD* = 0.68); responses were statistically equivalent to chance responding, *t*(329) = 3.86, *p* < 0.001, with 84.5% of participants choosing the scale midpoint on this item.^3^ Additionally, participants thought that it is similarly wrong to harm animals with human brain tissue and “normal” animals (*M* = 4.01, *SD* = 0.96); responses were statistically equivalent to the scale midpoint, *t*(329) = 4.01, *p* < 0.001, with 78.8% of participants choosing the midpoint on this item.

Additionally, participants thought that it was equally permissible to perform scientific experiments on rats with implants of human brain tissue (*M* = 4.80, *SD* = 1.76) as on “normal” rats (*M* = 4.75, *SD* = 1.76); responses on these two questions were statistically equivalent to each other, *t*(329) = 4.97, *p* < 0.001. Participants also thought it would be equally permissible to sacrifice chimeric rats (*M* = 3.25, *SD* = 1.95) and normal rats (*M* = 3.26, *SD* = 1.97). Responses to these two questions were statistically equivalent to each other, *t*(329) = 5.76, *p* < 0.001.

We further asked more directly about potentially blurred lines between humans and animals in the case of chimeras. Participants generally disagreed that implanting human brain tissue blurs the line between humans and animals (*M* = 2.01, *SD* = 1.00),^4^*t*(329) =  −36.18, *p* < 0.001, *d* = 1.99, again strongly casting doubt on Robert and Baylis’s central descriptive assertion. Furthermore, participants were generally not resistant to these scientific technologies (*M* = 3.17, *SD* = 1.53),^5^*t*(329) =  −9.88, *p* < 0.001, *d* = 0.54.

Finally, participants typically regarded animals with human brain tissue as being 0% human; this was the modal response given for rats (76.7% of participants), pigs (75.2% of participants), and chimpanzees (73.3% of participants). The average percentage (across the three items) was 3.78% (*SD* = 12.02%). In conclusion, in this more elaborate study on the moral status of animals containing human organs, we observed little to no perceived blurring between human and non-human animal kinds.

## Discussion

Recent scientific breakthroughs in xenotransplantation and human-animal chimera research have intensified discussions about the ethics of the creation and treatment of human-animal chimeras. In those discussions, arguments and claims often implicitly or explicitly rely on empirical assumptions about how people think about or will react to human-animal chimeras. We inform this debate by empirically testing some of these assumptions in three studies. Specifically, we sought to bring psychological data to bear on the issue of whether bioethicists can reasonably expect that chimeras will create a form of moral confusion amongst the general public.

Taken together, our three studies indicate that lay people find typical forms of xenotransplantation morally unproblematic. In our first study, people believed that the moral status of humans remains unchanged upon obtaining an organ transplant from an animal. Thus, when it comes to the moral status of humans with animal cells, tissues, or organs, there is no indication that people will be morally confused.

It is worth noting that our findings about public attitudes toward xenotransplantation may be influenced by how these technologies are presented in lay media. While current xenotransplantation involves specific genetic modifications (such as the addition of human genes to pig organs), media coverage often focuses on the transplant procedure rather than the genetic modification aspects. Future research could investigate whether more detailed understanding of the genetic modifications involved would affect public moral evaluations of these procedures.

What about the moral status of animals containing human cells, tissues, or organs? Here our evidence is more mixed. The results of Study 2 suggest the moral status people assign to animals is somewhat more flexible depending on whether the animals obtained human tissues or organs or not. People tend to accord a slightly higher moral status to pigs with human organs compared to standard pigs and a slightly higher moral status still to pigs with human brain cells compared to pigs with human organs. Results from Study 3, on the other hand, only partly replicated these findings. The study suggests that people do not accord a higher moral status to animals with human brain tissue, at least where there is no clear expectation that this will enhance their cognitive capacities. Perhaps most strikingly, most participants regarded animals (including chimpanzees) with human brain tissue as being zero per cent human.

That pigs with human brain cells were assigned a somewhat higher moral status than pigs with human organs might be explained by the tendency of lay people to accord moral status based not only on species membership but also, and independently, on cognitive capacity (Caviola et al. 2022). Thus, the pigs with human brain cells may have been assigned a higher moral status because participants thought that this may potentially lead to enhanced cognition; indeed, past research has suggested that people give greater weight to perceived cognitive capacity in the case of animals, while giving greater weight to species membership in the case of humans (Caviola et al. 2022). In Study 3, such potentially enhanced cognition was explicitly ruled out, and this arguably explains why animals with human brain tissue were not accorded a higher moral status. Importantly, however, though cognitive capacity has been found to be an independent contributor to moral status on lay views, it does not trump species membership, implying that, for example, a human with low cognitive capacities will typically be assigned a higher moral status than an animal with higher cognitive capacities (Caviola et al. 2022). Thus, even if people assigned a much higher moral status to a human-animal chimera with potentially *significantly* enhanced cognitive capacities, this need not at all threaten humans’ moral status (as those worrying about moral confusion fear), since for most people species membership trumps cognitive capacities as a criterion for moral status. In other words, as long as cognitively enhanced human-animal chimeras are still considered animals, confusion about their moral status is very unlikely.

Moreover, it is worth noting that animals’ cognitive capacities could potentially be enhanced in more direct ways that do not involve human-chimera technology (e.g., through genetic modification without using human cells). While our studies did not directly compare how people would view animals with enhanced cognition achieved through different methods, this raises an interesting question for future research: whether people focus more on the resulting cognitive capabilities or on how these capabilities were achieved. In our current findings, pigs with human neurons were still fundamentally regarded as animals, albeit with a slightly higher moral status because of their capacities. The elevation in moral status may be comparable to how people generally tend to accord a different moral status to animals based on their cognitive capabilities (e.g., placing chimpanzees above ostriches). It may be the potential for enhanced cognition, rather than the human origin of the enhancement, that drives these moral status judgements. This suggests that the human component of the chimeras does not seem to be doing any real work when people assign a higher moral status to pigs with human neurons.

The question remains, however, why pigs with human organs were being accorded a slightly increased moral status compared to normal pigs in Study 2 (though, again, animals with human brain tissue were not accorded a higher moral status compared to normal animals in Study 3). We know that lay people tend to accord moral status primarily based on species membership (i.e., belonging to the human species gives you the highest moral status), and Study 3 suggests that people think that animals with human tissue are zero per cent human. Thus, though animals’ moral status may be slightly elevated if they contain human tissues or organs, they are still categorically classified as animals. We found no evidence at all for confusion on this crucial point.

To the extent that the slight elevation in the moral status of pigs with human organs we found in Study 2 reflects a genuine phenomenon, it could perhaps be explained by the fact that people value humans and human cells, tissues, and organs above pigs (and animals in general) and their cells, tissues, and organs. So, though animals with human parts are not considered human, they have parts that make them more valued. This might be in line with the special moral significance widely attributed to human body parts—for example, that of the deceased—which is very different from that attributed to the body parts of animals.

Our studies focused on broadly realistic forms of animal-human chimeras of the kinds currently being developed or discussed. They cannot rule out that more radical forms of chimeras would cause genuine moral confusion—for example, chimeras involving animals consisting of far more human cells, tissues, or organs, which therefore might be more challenging to categorize as either human or animal. Future research could investigate this possibility. Likewise, when considering the ethics of xenotransplantation, people’s views may be affected by the number or sort of animal organs that are transplanted, and further work could investigate whether there is a tipping point at which a human who has an increasing number of animal organs is eventually perceived as less human. For example, In Study 1, we only tested for transplants of animal hearts and kidneys. It is certainly possible that the transplantation of an entire pig brain into a human body would be genuinely confusing, though it is also worth noting that people generally do not hesitate to ascribe full moral status to actual humans whose cognitive capacities are broadly similar to those of some animals. While the present studies do not cover these more radical possibilities, it remains the case that these currently belong to the realm of science fiction and are not particularly relevant to the current bioethical debate about the ethics of human-animal chimeras.

While this paper focuses on testing the empirical assumptions of the moral confusion argument, our findings have broader implications for xenotransplantation policy and implementation. There exists, however, a notable gap between public views and bioethical positions on animal moral status (Koplin 2023). This raises questions about how to weigh public attitudes in policy decisions, particularly when these may reflect unchallenged speciesist biases. While these questions about the relationship between public opinion and morally grounded policymaking are important, they require separate analysis beyond this paper’s scope.

Note also that while our studies examined attitudes toward xenotransplantation and human-animal chimeras more generally, we did not specifically investigate attitudes toward exotransplantation—the transplantation of primarily human organs grown in animals into human recipients. This type of transplantation might elicit different moral intuitions and reactions from the public. For example, people might view these organs as more human than the animal organs used in xenotransplantation, potentially raising different questions about moral status and species boundaries. Future research could specifically examine public attitudes toward exotransplantation.

It also bears mentioning that, across our three studies, the moral values of chimeric and non-chimeric individuals remained virtually identical, and the slight tendency we observed for animals to be enhanced in their abstract moral value by human (brain) tissue and organs did not translate into a more tangible difference in perceived rights or non-permissibility to harm. Thus, to the extent that the presence of human cells does somewhat alter the perceived moral status of animals, this effect might nevertheless have little to no practical import.

## Conclusion

Like other dramatic biomedical advances, the development of human-animal chimeras raises important ethical questions. One aspect of these questions relates to the way such chimeras would be perceived by the general public. Whether or not those public perceptions reflect robust ethical arguments, they can significantly affect the reception of those biomedical developments and lead to adverse consequences. Robert and Baylis have argued, in that vein, that human-animal chimeras will generate serious confusion about moral status and that this risk is a serious worry about continuing such biomedical research. It is important to recognize that this is an empirical claim, a claim that ultimately needs to be supported, or rejected, with empirical evidence. The studies reported here aimed to test this claim. Across three studies, we found no evidence that the public finds, or will find, human-animal chimeras confusing, or that they have the potential to challenge common conceptions of moral status.

## Supplementary Information

Below is the link to the electronic supplementary material.

Supplementary file1 (DOCX 24 KB)

## Data Availability

Data for all three studies are available at https://osf.io/j6vkr/.

## Footnotes

1 Nonetheless, participants did find xenotransplantation harmful to animals (*M* = 4.84, *SD* = 1.70, *p* <.001, *d* = 0.50), and they were ambivalent about whether this procedure is “unnatural” (*M* = 3.98, *SD* = 1.85, *p* =.809, *d* =  − 0.01).

2 Crucially, these questions asked people to make evaluations about “animals with human brain tissue implants” (with this term used in a generic sense and not indicating any particular species).

3 Equivalence tests provide a frequentist option for detecting the absence of a meaningful effect, by testing whether an effect is significantly lower than the smallest effect size of interest. For background on this approach, see Lakens, Scheel, and Isager 2018).

4 This composite variable was created by averaging across three items: “Implanting rats with human brain tissue also imparts them with a human ‘essence’,” “If human brain tissue that had been implanted into a laboratory rat were to later be transplanted into a person who was suffering from a neurological condition, that person would become less human,” and “If a chimpanzee were to receive a human brain tissue implant (in a way that didn’t impact the chimpanzee’s psychological functioning), this would blur the distinction between animals and humans” (α =.74).

5 This composite variable was created by averaging across four items: “I feel grossed out by thinking about rats with human brain tissue,” “Scientists should not implant human brain tissue into rats under any circumstances,” “If I needed to have an implant of brain tissue for medical reasons, I would be OK with getting an implant from a pig” (reverse scored), and “If I needed to have an implant of brain tissue for medical reasons, I would be OK with getting an implant from a chimpanzee” (reverse scored) (α =.88).
