# Supplementary material for: Will Human-Animal Chimeras Cause Moral Confusion? Exploring Public Attitudes
Source: J Bioeth Inq. 2025 Aug 12;22(3):733–44. doi: 10.1007/s11673-024-10413-4 (PMC12575588; doi:10.1007/s11673-024-10413-4)
Supplement: Supplementary file 1 — Supplementary file1 (DOCX 24 KB) [file 11673_2024_10413_MOESM1_ESM.docx]

# Supplementary Materials

**“Will Human-Animal Chimeras Cause Moral Confusion: Exploring Public Attitudes”**

## Study 1

### Study procedure

Participants read the following text in the beginning:

“Please read the following text carefully before you proceed:

“As science and technology progress, it is becoming increasingly possible and safe to transplant cells, tissues, or organs from non-human animals to humans. Earlier this year, doctors at the University of Maryland transplanted a pig heart into an adult human with terminal heart disease. The heart came from a pig that was specially bred in order to prevent immunological rejection (and many other medical issues that could arise out of such a transplantation procedure), and indeed, the patient had no rejection response weeks after the surgery. With that, a first step to the success of such procedures has been taken. Similarly, scientists recently transplanted two pig kidneys to a patient who was already brain-dead. (His family authorized the procedure.) The kidneys were found to function and were not rejected, which is promising for transplanting pig kidneys to non-brain-dead patients in the future. In sum, it is becoming increasingly possible to successfully transplant various types of organs from animals in order to improve the health of human recipients.”

Participants answered the following comprehension questions. They must answer all correctly to proceed:

“What did you learn from the text on the previous page?”

1. It is becoming increasingly possible to transplant animals’ body parts to human beings. (**Yes**/No)
2. Doctors have transplanted a cow’s heart into a human. (Yes/**No**)
3. Doctors have transplanted a pig’s heart into a human. (**Yes**/No)
4. Doctors have transplanted a pig’s kidney into a human. (**Yes**/No)
5. Doctors have transplanted a cow’s kidney into a human. (Yes/**No**)

Participants could attempt these questions for a second time if they did not answer all correctly on their first try. In that case, they would be presented with the introduction text again. If they still failed, they were not allowed to proceed. Participants completed measures detailed in the following section, reported demographic information (age, gender identification, education status, estimated social class, and English language proficiency), and were thanked and debriefed in the end.

### Measures

Participants were randomly assigned to two conditions. In one, they reported their attitudes about transplanting animal hearts to humans; in the other, they reported their attitudes about animal kidneys. We included the following attitude measures:

Evaluation of xenotransplantation

1. **Moral judgments of xenotransplantation** (the general question first, then the specific question, which is presented on a separate page; both on −3 = very wrong, 3 = very right):
   - General: “How morally right or wrong is transplanting animal organs to humans?”
   - Specific: “How morally right or wrong is transplanting ____ to humans?” (animal hearts or animal kidneys, depending on the condition participants are in)
2. **Reasons for moral wrongness**: “There can be different reasons why people might find transplanting an animal heart/kidney to a human problematic. To what extent do you agree with the following statements?” (1 = Strongly Disagree, 2 = Disagree, 3 = Somewhat Disagree, 4 = Neither Agree nor Disagree, 5 = Somewhat Agree, 6 = Agree, 7 = Strongly Agree)
   - “Transplanting animal hearts/kidneys into humans makes me feel nauseated.”
   - “It harms animals to harvest their hearts/kidneys for humans’ use.”
   - “Transplanting animal hearts/kidneys into humans is unnatural.”
   - “Transplanting animal hearts/kidneys into humans is medically unsafe.”
   - “Transplanting animal hearts/kidneys into humans is against God’s will.”
   - “Transplanting animal hearts/kidneys into humans makes the recipient human impure.”
   - “Transplanting animal hearts/kidneys into humans blurs the distinction between humans and animals.”

Influence on xenotransplant receivers

“Please indicate your agreement with the following statements.” (−3 = strongly disagree, 3 = strongly agree)

1. Species identity [1]: “Having an animal heart/kidney in one’s body would make the person less human.”
2. Species identity [2]: “Having an animal heart/kidney in one’s body would make the person somewhat similar to that animal.”
3. Moral worth: “Having an animal heart/kidney in one’s body would make the person less morally valuable.”
4. Agency [1]: “Having an animal heart/kidney in one’s body would change how the person thinks and reasons.”
5. Agency [2]: “A person with an animal heart/kidney in their body would be less responsible for their behaviors.”
6. Experience: “Having an animal heart/kidney would cause a person to have more animal-like sensory and emotional experiences.”
7. Behavioral intention: “I would consider receiving an animal heart/kidney if it would save my life.”
8. “If I received an animal heart/kidney, I would see it as part of me.”
9. “If you would need a transplant and could choose between an artificial organ (one made out of unnatural materials) and an animal organ, which would you choose if they were equally safe and functioning?” (−4 = definitely the artificial organ, 4 = definitely the animal organ)

Participants also completed the following scales:

1. 6-item Speciesism Scale (Caviola et al., 2019)
2. 9-item Oxford Utilitarianism Scale (Kahane et al., 2018)
3. Religiosity (7-point scale): “
4. Economic and social conservatism (one item each; 7-point scale)
5. Nature bias
   1. Preference for natural products
   2. Preference for environmental preservation
   3. Against interventions to the human body
   4. Against human enhancement
   5. Against intervention in nature
   6. Science skepticism
6. Disgust Scale-Revised (Olatunji et al., 2007)
7. Bastian and Haslam’s (2006) 23-item scale for psychological essentialism
8. Ecological Dominance Orientation (Uenal et al., 2022)
9. Human supremacy beliefs (Dhont & Hodson, 2014)

## Study 2

The survey comprised of four sections. The first section consisted of questions adapted from Sawai et al.’s 2017 study in order to perform a cross-cultural comparison between the UK and the Japanese publics’ attitudes on human-animal chimeric embryo research. The results from this section are intended for another publication, and will not be discussed further here.

In the second section, participants were asked the questions that are reported in the main text. Variables included the type of organ from the other species (brain cells, heart, liver and facial features), and having either one or two organs from the other species. A between-subjects design was employed in which participants were randomly allocated to either the pig or human condition. This experimental design was chosen to avoid order effects such as practice or fatigue effects. All questions were randomised to avoid order-effect bias.

The third section aimed to assess participants’ views on the moral status of pigs and pigs with human organs with disabled cognitive capacities. Participants were asked how much they morally value pigs that lacked the ability to feel pain or consciousness when compared to a standard pig, as well as how much they morally value pigs with human organs that lack the ability to feel pain or consciousness when compared to a non-disabled pig with human organs. Again, participants were asked to answer on a comparative scale where 1 was ‘Much less’, 4 was ‘Exactly the same’ (as a standard pig or a non-disabled pig with human organs), and 7 was ‘Much more’. Section Three also aimed to further explore previously published public concerns over the humanisation of animal brains by asking participants whether they believed that creating pigs with human organs which were more intelligent than a standard pig was ethically permissible. Furthermore, because we hypothesized that participants would find the creation of pigs with human organs that were more intelligent than a standard pig to be wrong, participants were then asked whether they believed that genetically modifying pigs with human organs to be no more intelligent than a standard pig, to not be able to feel pain, or to lack consciousness was ethically permissible. Participants were asked to indicate to what extent they agreed on a seven-point Likert scale where 1 was ‘Very wrong’, 4 was a neutral point of ‘Neither wrong nor right’, and 7 was ‘Very right. Again, all questions were randomised to avoid order-effect bias.

In Section Four, participants were asked to complete the Speciesism and Oxford Utilitarian Scales (OUS) that were also used in Study 1, and to provide demographic data (gender, age, ethnicity, education level, income level, social class, religious affiliation, political affiliation, and vegetarian or vegan lifestyle).

To improve the validity of the survey data by ensuring that participants were paying attention to questions before answering them, attention checks were employed throughout the survey. The first attention check in Section One was aimed at ensuring respondent attention and comprehension (“Based on the information we just provided, researchers are trying to produce which human organ in pigs?”). The other three attention checks were spread throughout the survey and were designed to check that participants were paying attention and had read the text (e.g. “Please selection the second option from the left.”).

## Study 3

In addition to the primary measures, participants additionally completed scales assessing their tendencies to value humans above all other animals (“Speciesism Scale”; Caviola, Everett and Faber 2019), their tendencies to value humans more than nature (“Tree-Hugger vs. Human-Lover Scale”; Rottman, Crimston and Syropolous 2021), their preferences for hierarchical relationships between humans and animals (“Ecological Dominance Orientation”; Uenal et al. 2022), and their desires for certainty and predictability (“Need for Closure Scale”; Roets and Van Hiel 2011).

We regressed each of these four individual differences measures onto each of the nine primary variables described in the main text. Across these nine regression analyses, we uncovered few robust patterns. Speciesism negatively predicted two of the three moral standing variables––attributing greater moral rights to chimeras (*b* = -0.10, *p* = .004) and thinking it is more wrong to harm chimeras (*b* = -0.24, *p* < .001)––as well as resistance to xenotransplantation (*b* = -0.35, *p* < .001). Ecological Dominance Orientation predicted being more willing to eat bacon from a normal (as compared to chimeric) pig (*b* = 0.19, *p* = .004) as well as thinking that chimeras are more human (*b* = 1.46, *p* = .001). Being a “Tree-Hugger” rather than a “Human Lover” positively predicted perceptions that xenotransplantation blurs the line between human and non-human animals (*b* = 0.24, *p* < .001). Need for Cognition did not uniquely predict any variables.
